# The sights and insights of examiners in objective structured clinical examinations

**DOI:** 10.3352/jeehp.2017.14.34

**Published:** 2017-12-27

**Authors:** Lauren Chong, Silas Taylor, Matthew Haywood, Barbara-Ann Adelstein, Boaz Shulruf

**Affiliations:** 1 Clinical Skills Teaching Unit, Prince of Wales Hospital, Sydney, Australia; 2 Office of Medical Education, University of New South Wales, Sydney, Australia; 3 University of New South Wales, Sydney, Australia; 4 Prince of Wales Clinical School, University of New South Wales, Sydney, Australia; 5 Centre for Medical and Health Sciences Education, University of Auckland, Auckland, New Zealand; Hallym University, Korea

**Keywords:** Bias, Leadership, MEDLINE, Problem solving, Student

## Abstract

**Purpose:**

The objective structured clinical examination (OSCE) is considered to be one of the most robust methods of clinical assessment. One of its strengths lies in its ability to minimise the effects of examiner bias due to the standardisation of items and tasks for each candidate. However, OSCE examiners’ assessment scores are influenced by several factors that may jeopardise the assumed objectivity of OSCEs. To better understand this phenomenon, the current review aims to determine and describe important sources of examiner bias and the factors affecting examiners’ assessments.

**Methods:**

We performed a narrative review of the medical literature using Medline. All articles meeting the selection criteria were reviewed, with salient points extracted and synthesised into a clear and comprehensive summary of the knowledge in this area.

**Results:**

OSCE examiners’ assessment scores are influenced by factors belonging to 4 different domains: examination context, examinee characteristics, examinee-examiner interactions, and examiner characteristics. These domains are composed of several factors including halo, hawk/dove and OSCE contrast effects; the examiner’s gender and ethnicity; training; lifetime experience in assessing; leadership and familiarity with students; station type; and site effects.

**Conclusion:**

Several factors may influence the presumed objectivity of examiners’ assessments, and these factors need to be addressed to ensure the objectivity of OSCEs. We offer insights into directions for future research to better understand and address the phenomenon of examiner bias.

## Introduction

The objective structured clinical examination (OSCE), introduced by Harden in 1975, is considered to be one of the most robust methods used for clinical assessment across medicine, nursing, exercise physiotherapy, and allied health programs [1-3]. It is most commonly used for summative, high-stakes assessments in medicine, nursing, and clinical psychology education programs [1,2,4,5], and as a selection tool for training and licensure for practice [1,6,7]. An OSCE requires each student to demonstrate specific skills and behaviours, typically in a series of short assessment tasks (stations), each of which is assessed by an examiner using a predetermined objective marking scheme [2]. Whilst OSCEs vary in their specific requirements and process across jurisdictions, the overall design of the OSCE has traditionally been viewed as advantageous, as it standardises the items and tasks for each candidate. Consequently, it has also been considered to minimise the effects of examiner bias through the use of ‘identical’ patients, structured checklists, and multiple assessor-candidate interactions across a number of stations [1,8]. Despite the intention of this design, OSCEs are in practice prone to high levels of variance [9]. Under ideal circumstances, scores should vary only as a reflection of student capability; however, the evidence shows that a key source of variability originates from the examiner [10-12]. Such examiner effects include assessor stringency or leniency, the halo effect, and a range of pre-existing biases [13,14]. Indeed, up to 29% of score variation may be explained by examiner stringency alone [14,15].

To ensure the validity of the OSCE as an assessment tool, it is crucial to understand and evaluate these sources of examiner bias [6,7]. Traditionally, research in medical, nursing, and allied health education has focused on the reliability of assessments (e.g., items’ internal consistency or inter-rater agreement), with less attention given to the effect of examiners’ biases on the validity of the assessment [16]. Contemporary studies, however, have focused more on assessors’ personal attributes and the nature and validity of the assumptions by which they are guided, and which eventually affect their judgment and scoring [17,18]. This critical review aims to discuss sources of examiner bias, and offers insights into directions for future research to better understand and address this phenomenon.

## Methods

### Study design

This systematic review used the Preferred Reporting Items for Systematic Reviews and Meta-Analyses (PRISMA) guidelines.

Literature search process: We searched the medical literature using Medline (1946–April 2017) between January and April 2017 for papers that addressed the topic of examiner bias in OSCE settings. Search strategy were as follows:

1. OSCE.mp.
2. Objective structured clinical exam.mp.
3. 1 or 2
4. Bias.mp.
5. 3 and 4
6. Assessor bias.m_titl.
7. Examiner bias.mp.
8. 6 or 7
9. 3 and 8
10. Halo effect.mp.
11. Hawk dove.mp.
12. Hawk dove effect.mp.
13. 11 or 12
14. Examiner fatigue.mp.
15. 1 and 14
16. 5 or 8 or 10 or 13 or 14

**Fig. 1.**
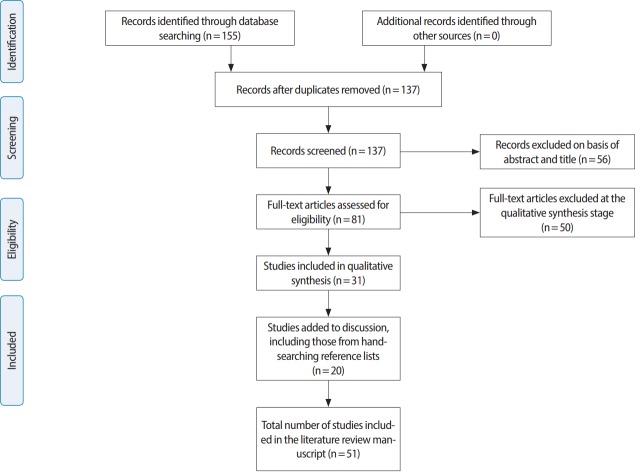
PRISMA (preferred reporting items for systematic reviews and meta-analyses) flow diagram. From Moher et al. PLoS Med 2009;6:e1000097 [19].

No restrictions were initially placed on the publication date within our search, although we only included publications within the last decade in our final analysis, resulting in the exclusion of any results pre-dating this period. Appropriate articles (n= 51) were reviewed, and salient points were extracted and synthesised into a clear and comprehensive summary of the knowledge in this area [19] (Fig. 1). LC conducted the initial screening of titles and abstracts and excluded articles that did not fulfil the inclusion criteria. The full texts of the remaining articles were independently reviewed by 2 authors (LC and BS), and studies that met the eligibility criteria were used in the final synthesis. Any discrepancies were resolved via discussion with the author team.

## Results

### Internal factors affecting OSCE examiners

#### Halo effect

One of the most studied types of rater effects is the ‘halo effect’ [7,13,20-22]. The halo effect is a cognitive bias in which an assessor fails to discriminate among independent aspects of behaviour when making a judgement about a student [7,22]. For example, the halo effect may occur when an examiner makes a judgement based on a general impression, such as a first impression, which then influences all subsequent judgements or ratings. Another example of the halo effect occurs when a rater allows an individual’s performance in one domain, such as communication, to influence judgements of his or her performance in other domains. This effect is a threat to the validity of inferences made based on performance ratings, as it produces inappropriately similar ratings across items [20].

#### Hawkishness and dovishness

A potential vulnerability of any clinical examination is that examiners differ in their relative leniency or stringency. This is often termed the ‘hawk-dove’ effect [14]. Hawks tend to fail more candidates because of having very high standards; doves tend to pass more candidates due to greater leniency. The effect arises from examiners’ own perceptions of the standards required for the exam, as well as from personality factors. Variance as high as 45% due to examiner stringency or leniency has been reported, thus making the hawk-dove effect one of the most significant factors influencing student outcomes [9]. In this study, a shift of 11% of OSCE candidates across the pass/fail line was demonstrated when the examiner stringency/leniency effect was removed from communication scores in a 6-station OSCE. At the ends of the examiner leniency distribution curve lie the ‘extreme’ assessors, defined as individuals giving a mean score greater or less than 3 standard deviations above or below the collective mean score [23]. The extreme nature of their assessments may be due to individual characteristics of an examiner, or less commonly, simple marking errors, for example grading 1/5 as ‘excellent’ and 5/5 as ‘fail’ when the opposite is correct [8].

#### Examiner demographics

Examiner sex and ethnicity were found not to predict score variance among general practitioner trainees in a clinical skills assessment, a finding supported by a similar study showing that examiner demographics (gender, UK or international medical degree, white or other background) explained only 0.2% of performance variance [24,25]. The level of training of the examiner likewise does not affect stringency or leniency [9]; however, both trained and untrained assessors tend to be more lenient and award higher marks to female students, although this interaction may only be slight and not statistically significant [20,26]. The influence of student-patient-examiner gender composition on examiner scores has not been reported, despite evidence from Australian medical schools that the opportunity to practice physical examinations on the opposite gender is limited [27]. Nonetheless, in the specific domain of communication skills assessment, a tendency exists for female students to perform significantly better than males [28]. This may be due to a combination of innately superior communication abilities in females, as well as gender interactions among the student, patient, and examiner. It has been shown that simulated patients tend to rate female students higher in communication skills than males through an effect independent of their own gender [29-31], and, while relatively little data exist on the effect of examiner and student gender interactions, Schleicher et al. [32] reported that male examiners awarded significantly higher communication skills ratings to female examinees.

Despite the above findings, the literature is still not entirely clear. Writing in 2013, Esmail and Roberts [33] commented that “we (cannot) confidently exclude bias from the examiners in the way that they assessed non-white candidates.” While it is recognised that students from certain ethnic minorities may perform more poorly on assessments independent of any examiner bias, it is possible that examiner variance may also be up to 4 times greater than that of examinees [9,34]. Concern around issues such as these was sufficiently important to instigate the development of a cultural competence training module at Harvard Medical School [35]. A possible explanation for greater stringency is that people from individualistic cultures such as North America or Western Europe tend to place a higher value on personal independence, whereas people from collectivist cultures such as Asia, the Middle East, or some indigenous groups focus more on interdependence and relatedness to the community [36]. Thus, the latter may be more influenced by ‘leadership’ bias when multiple examiners are present, adopting the more stringent approach associated with examiners of greater clinical or assessment experience, who are also normally the more senior amongst the OSCE panel members [17]. The effect of a doctor’s background on clinical practice has been recognised among international medical graduates who undergo a difficult acculturation process to both the general culture and the healthcare subculture in their host country [37].

#### Duration of examining during an assessment period

Students sitting an OSCE station early in the day receive higher marks on average than those sitting it later [6]. For example, Hope and Cameron [6] found a difference of 3.27% in marks between the first and last students sitting a station during a day, and it was predicted that 2 failing students would have passed had they been assessed in the morning. While this effect is small, it may impact students close to the pass/fail borderline or those in contention for awards. Variation by time of day has been attributed to examiner fatigue as the OSCE continues, as opposed to examiner ‘warm-up’ in the first few stations [1]. In contrast, some evidence suggests that increasing examiner fatigue over time leads to reduced attention to student errors and thus a tendency to award higher scores later in the day, even when adjusting for the warm-up phenomenon [38]. With regard to prolonged periods of OSCE assessment, assessors tend to be more lenient at the start and become more hawkish with time [6].

#### The contrast effect

Assessors tend to judge performance comparatively, rather than against fixed standards [39]. They tend to mentally amalgamate previous performances, especially those seen early on, to produce a performance standard to judge against. Thus, examiners who have recently observed and scored good performances give lower scores to borderline candidates than those who recently observed and scored poor performances [6,9,39]. This effect occurs across different parts of the educational curriculum, in non-clinical and clinical exams, different geographical locations, and different formats of examiner response (behavioural and global ratings) [40]. Examiners also show a lack of insight into their susceptibility to this phenomenon [39]. Anchoring bias (originally discussed in the context of diagnostic reasoning) is related to contrast bias and can be regarded as the influence of recent experiences on the examiner’s subsequent ratings [41]. The examiner may ascribe disproportionate significance to certain features if exhibited by multiple examinees, thus leading to the award of a higher grade than a candidate deserves if he or she is preceded by a good performance. Hawkishness and dovishness are influenced in a similar way by the performance of recently observed candidates at any level, although the impact of this is less than that of the contrast effect [1,6].

#### Training of examiners and lifetime experience in assessment

Untrained assessors, as well as those with limited involvement in exam construction, award higher marks than trained assessors [6,13,42]. This may be attributable to a lack of understanding of the rating criteria and a poorer appreciation of the exact purpose, format, and scoring of the assessment [13,21]. Assessor training is therefore arguably an important component of a valid OSCE, as experienced examiners may set higher pass thresholds in OSCEs at least partially as a result of their greater confidence with the marking scheme or understanding of student standards [6,43]. Assessors may also use themselves as a reference point, leading to harsher candidate ratings as they become more experienced. Training is therefore important for both novice and experienced assessors in an attempt to ensure consistency across examiners.

#### Physician versus non-physician examiners

Good agreement exists between physicians and trained non-physician examiners when scoring against checklists [44]. However, there is poor agreement on pass/fail decisions, and up to 25% of students are misclassified by trained non-physician assessors, suggesting they are not as competent in completing global rating scales as trained physician examiners. This may be because non-physicians lack the medical knowledge to give credit to certain lines of questioning, such as those that ask the candidate to rule out certain differential diagnoses. However, it is interesting to note that among physician examiners, familiarity with a speciality does not influence the marks awarded [45].

#### Leadership and familiarity with students

If multiple examiners are present, they are influenced by the scores awarded by those with greater expertise or the perceived ‘leader’ [17]. Furthermore, examiners who are familiar with the students are more generous than those who are not [6,13]. This latter phenomenon may be a product of the ‘mere exposure effect’ whereby individuals favour things familiar to them [6].

### External factors affecting OSCE examiners

#### Station type

A weak and statistically insignificant relationship was found between examiner scoring and the content area being examined [1]. Communication stations, such as taking a history or breaking bad news, may involve less assessor interaction than clinical examination stations [1]. This may increase the likelihood of assessor fatigue and disengagement, resulting in a higher or lower score than warranted by the performance. Some assessors are also less familiar with communication skill stations than with physical examination skill stations, but training in grading the former has been shown to reduce inter-rater variability [46]. Although the station type may produce bias in OSCE marks, station difficulty and order do not [6,47]. An ongoing tension exists between OSCE performances as determined by global rating scores and more objective, itemised checklist scores, particularly for borderline students [48]. When global and checklist scores are employed within a single station, some evidence indicates that assessors use different traits to inform their impression of these 2 metrics, perhaps due to inadequate assessor training or different levels of experience [48].

#### Site effect

This multifactorial source of bias is complex and not easily categorised under any of the above domains; however, it is recognised that different medical schools would not award the same score to an identically performing student at an identical OSCE station [18]. Differences in the agreed pass score, scoring criteria, simulated patient behaviour and examiner behaviour, and training have all been implicated and may even be inter-related. For example, a simulated patient’s conduct may affect the student’s performance directly, as well as influencing the examiner’s perception of that performance. Similarly, the local choice of statistical analysis will also influence the proportion of students passing an OSCE. A comparison of 2 statistical analyses on the same data set demonstrated that the borderline regression method resulted in a higher pass mark and a larger difference margin in the failure rate than another common method when analysing smaller groups of students [49].

## Discussion

Overall, this comprehensive (but not fully systematic) review identified several factors influencing OSCE examiners’ assessment scores. The psychology and impact of the halo [7,13,21,22] and hawk/dove effects [1,9] are well understood, but further research is required into the influence of the contrast effect (and its duration) and the examiner’s gender, ethnicity, training, lifetime experience in assessing, leadership, and familiarity with students. In addition, little is known about the effect of the assessment type (e.g., formative or summative), marking criteria, and exam tasks on examiners’ judgements [6,13].

The authors propose that the factors discussed in this paper can be categorised into 4 major domains: examination context, examinee characteristics, examinee-examiner interactions, and examiner characteristics. Table 1 summarises the factors that are likely to raise the marks of an OSCE examinee. It should be noted that additional factors may influence the level of error (e.g., whether the examiner is a clinician), but no evidence of bias has been found.

An improved understanding of the potential role of these factors is crucial to reassure candidates and employers of the validity of OSCEs. This is especially true in a time of increased scrutiny surrounding health professional examinations [1]. Addressing these concerns will also have important implications for students close to the pass/fail borderline and those in contention for awards [6,9].

While this review comprehensively summarises the biases in OSCE that are known to exist, the next step for researchers is to establish why they exist. Attempts to address examiner subjectivity through measurement standardisation have been largely unsuccessful [50,51], resulting in the recent emergence of rater cognition as a new field of research [52]. It is increasingly understood that assessors are motivated differently and form impressions of candidates dependent upon social interactions and context [51]. Variation in factors such as individuals’ concepts of competency, definitions of critical performance aspects, synthesis of information gleaned from observation, production of narrative assessments, and conversion into rating scales are all thought to be key variables that have hitherto received relatively little attention [52]. The challenge is therefore to move away from a focus on rating instruments and raters to a focus on the context of performance assessment, such that assessor cognition can be more fully understood and targeted as part of an ongoing effort to reduce bias.

### Limitations

Since this is not a classical systematic review, the authors cannot guarantee the comprehensiveness of the conclusions drawn in this paper. However, medical education is an evolving field and all contemporary evidence was evaluated. Medline contains more than 24 million references to life sciences and biomedical journals, and thus we argue that any relevant publication that was omitted from this paper as a result of not being indexed in Medline is unlikely to represent a substantial body of research not already discussed above.

### Factors influencing OSCE examiners’ assessment score

Once a stronger understanding of these issues is attained, strategies can then be implemented to address them. However, the challenge will be to achieve a suitable balance once interventions to remedy such biases are put in place. In other words, what does an optimal OSCE look like? We believe that the answer to this question is mostly not to be found within the statistical or psychometrical domains. All statistical analyses and psychometric techniques rely on the data generated by examiners who observe a performance and process that observation with their own skills, knowledge, prejudices, beliefs, and ability to accurately translate their decision into a predefined response or mark [36]. Thus, we urge future researchers to focus on the examiners’ cognitive processes during OSCEs [16], an area that hopefully will shed more light on this ‘black box’ of decision-making and improve our confidence in the well-established OSCE.

## Figures and Tables

**Table 1.** Factors likely to raise OSCE marks

Domain	Specific factors increasing the OSCE score
Examination context	Being examined at the beginning of the OSCE day
	Being examined after a poor examinee
Examinee characteristics	Female gender
	Having pre-existing good interpersonal skills
Examinee-examiner interaction	Previously acquainted with examiner
	Culturally matched
Examiner characteristics	Inexperienced or non-expert
	Similar rank/status to the examinee
	“Dove” (rather than “hawk”) inclination

OSCE, objective structured clinical exam.

## Supplementary material

Supplement 1. Audio recording of the abstract.
